# The effect of cerebral hypoperfusion on daily rhythms of locomotor activity in mice after carotid artery stenosis

**DOI:** 10.1002/alz70855_100874

**Published:** 2025-12-23

**Authors:** Elinor E Washington, Cathryn A Cutia, Mary E Harrington

**Affiliations:** ^1^ Smith College, Northampton, MA, USA

## Abstract

**Background:**

Cerebral hypoperfusion occurs when the flow of blood to the brain is decreased, resulting in inadequate blood flow to certain regions of the brain, potentially causing changes in circadian rhythms. The relationship between circadian rhythms and cerebral hypoperfusion has been studied in rats, but has not yet been studied in a mice model. Changes in circadian rhythms are associated with increased risk of developing Alzheimer's disease (AD).

**Method:**

In this study, we were also interested in seeing the effects of decreased blood flow on the corpus callosum, since deterioration in white matter tracts is seen in AD. The bilateral carotid artery stenosis (BCAS) procedure has been used to study vascular related diseases in mice models through restricting blood flow by wrapping a piano‐wire coil around the carotid artery. This procedure can also be performed unilaterally, placing a singular coil on either the right or left carotid artery. Locomotor activity (LMA) was recorded in a 12:12 light/dark cycle in C57Bl6/J male and female 6 month old mice before and after the surgery to determine if cerebral hypoperfusion has an effect on diurnal rhythms.

**Result:**

Our results indicate that LMA rhythms were maintained and were not affected by the BCAS surgery in both BCAS and sham mice, demonstrating that cerebral hypoperfusion does not disrupt diurnal rhythms in wildtype mice. After reducing blood flow of the BCAS mice, we aimed to further discover if white matter was decreased in the corpus callosum bilaterally or unilaterally depending on the number of coils situated during the BCAS surgery. In order to visualize changes in the white matter that result from reduced blood flow to the brain, the corpus callosum will be examined using Kluver‐Barrera staining and imaged for changes in myelination. Luxol fast blue was used to stain white matter tracts in coronal sections of both sham and BCAS mice.

**Conclusion:**

Our results indicate that cerebral hypoperfusion through BCAS might not impact diurnal LMA rhythms in wildtype mice.

